# Bridging the Gap: How Organ‐on‐a‐Chip Technology Facilitates the Battle against Glioma

**DOI:** 10.1002/smsc.202500154

**Published:** 2025-06-03

**Authors:** Su Liu, Zhenyu Gong, Dairan Zhou, Vanesa Ayala‐Nunez, Tao Xu, Peter Wick

**Affiliations:** ^1^ Nanomaterials in Health Laboratory Swiss Federal Laboratories for Materials Science and Technology (Empa) St. Gallen 9014 Switzerland; ^2^ Department of Health Sciences and Technology Eidgenössische Technische Hochschule Zürich (ETH) Zurich 8092 Switzerland; ^3^ Department of Neurosurgery Klinikumrechts der Isar Technical Uinversity of Munich 81675 Munich Germany; ^4^ Department of Neurosurgery Shanghai Changzheng Hospital Naval Medical University Shanghai 200003 P. R. China

**Keywords:** clinical impacts, glioma, glioma‐on‐a‐chip models, microfluidic devices, preclinical models

## Abstract

Glioma, a highly aggressive brain tumor, presents significant challenges in understanding its complex pathophysiology and developing effective treatments. This review critically examines the current glioma modeling platforms, including 2D culture, 3D cultures, glioma‐on‐a‐chip (GoC) models, and animal models, with a focus on their applications in recapitulating the tumor microenvironment and evaluating treatment effectiveness. Particular attention is given to microfluidic GoC models, which offer unique capabilities to mimic the pathophysiological complexity of glioblastoma, including its interactions with the blood–brain barrier (BBB) and tumor vascularization. Recent advancements in these models are explored, highlighting their potential for clinical translation through improved understanding of drug permeability on the central nervous system, tumor progress, and therapeutic responses. In the end, the review discusses the clinical perspective of GoC applications and establishes key criteria for designing robust and clinically relevant glioma models. By addressing these challenges and highlighting future opportunities, the critical role of GoC platforms in bridging the gap between preclinical research and clinical outcomes is emphasized, offering a promising approach for more personalized and effective glioma therapies.

## Graphical abstract

This review critically evaluates current state‐of‐the‐art *in vitro* glioma models especially glioma‐on‐a‐chip models based on readout capability, performance, and physiological relevance. It summarizes the key parameters and proposes a practical framework to guide researchers in selecting the most suitable preclinical models for glioma studies.

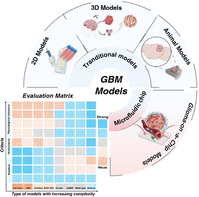

## 1 Introduction

Gliomas constitute a complex group of primary brain tumors originating from glial cells that surround and support neurons in the central nervous system (CNS). Among these, glioblastoma multiforme (GBM) stands out as the most aggressive and lethal form, classified as a Grade IV tumor by the World Health Organization (WHO).^[^ ^1^ ^]^ These tumors are notorious for their rapid progression, resistance to conventional therapies, and poor prognosis, with median survival times postdiagnosis typically not exceeding 15 months, even with optimal treatment strategies that include surgical resection, radiotherapy, and chemotherapy.^[^ ^2^ ^]^

The biological behavior of glioma is profoundly influenced by their cellular heterogeneity and the intricate tumor microenvironment.^[^ ^3^, ^4^, ^5^, ^6^ ^]^ These tumors are characterized by a robust proliferative capacity, high invasiveness, and substantial angiogenic potential, which together facilitate their expansion within the highly restrictive spaces of the brain.^[^ ^7^, ^8^ ^]^ Furthermore, gliomas demonstrate a remarkable ability to modulate their microenvironment, thereby promoting tumor survival and complicating therapeutic efforts. They achieve this through mechanisms such as the induction of local immune suppression, recruitment of vascular support via the secretion of angiogenic factors, and the manipulation of extracellular matrix (ECM) components to facilitate tumor invasion and dispersal.^[^ ^7^, ^9^, ^10^ ^]^

Moreover, the location of gliomas within the brain further complicates surgical interventions and limits the efficacy of therapeutic options.^[^ ^11^ ^]^ The diffuse nature of glioma margins often makes complete surgical resection impossible without risking severe neurological deficits.^[^ ^12^ ^]^ This is compounded by the tumor's capacity to infiltrate deeply into surrounding brain tissue, rendering local therapies less effective and often leaving residual tumor cells that lead to recurrence.^[^ ^13^ ^]^

The intricate biology and aggressive nature of glioma necessitate research models to unravel the complexities of tumor behavior and facilitate the development of novel treatment strategies. While GBM is a subtype of glioma, it is particularly notorious for its resistance to treatment and its ability to infiltrate healthy brain tissue. Because of these characteristics, this review emphasizes GBM especially in the context of developing more clinically relevant tumor‐on‐a‐chip models.

Traditional 2D cell culture models, although useful for high‐throughput drug screening and initial genetic profiling, significantly oversimplify the tumor environment and fail to predict clinical outcomes accurately due to their lack of 3D architecture.^[^ ^14^, ^15^ ^]^ To address these limitations, 3D tumor models have been developed to replicate crucial aspects such as cell‐cell and cell‐matrix interactions, spatial structure, gradients of oxygen and nutrients, and drug penetration.^[^ ^16^, ^17^, ^18^ ^]^ 3D glioma model includes spheroids generated from immortalized cell lines and organoids derived from patient tumor tissue or cancer stem cells. Compared to spheroids, elaborated by using cell lines, tumor organoids mimic more closely the real tumor architecture and patient heterogeneity because it is generated directly from a small piece of tumor biopsies. These models allow us to gain a better understanding of the complex behaviors of glioma, such as their invasive potential and resistance to therapies as well as can aid in developing more effective treatment strategies.

Further enhancing the realism in model systems, animal models, particularly genetically engineered mouse models and xenografts, have been instrumental in studying glioma growth within a living organism.^[^ ^19^ ^]^ These models allow for examining complex interactions between the tumor and its host environment and are invaluable for evaluating the efficacy and safety of new therapeutic agents.^[^ ^20^ ^]^ However, despite their utility, animal models are limited by high costs, increasing ethical concerns, and biological differences between humans and mice. These differences were the reason for the rather slow and time‐consuming translation of findings to clinical settings, as the response of human to a treatment may not be accurately predicted by the response of the mice model. This limitation underscores the need for more human‐relevant models, such as organ‐on‐chip models, which can offer a more accurate representation of human pathophysiological conditions. It should also be emphasized that since 2022, it is not essential anymore to test all new drugs in animal models to get the approval of the US Food and Drug Administration (FDA) based on the FDA Modernization Act 2.01342. This change accelerates the research in and future use of organ‐on‐a‐chip technology. In addition, a roadmap for organ‐on‐a‐chip models has recently been published, aiming to enhance their reproducibility and facilitate standardization.^[^ ^21^ ^]^ Therefore, the GoC model is a potential complementary approach to animal testing for new drug approvals concerning glioma. However, it is crucial to be aware of the remaining challenges and limitations of these novel *in vitro* models for optimal use.^[^ ^22^ ^]^

Emerging at the forefront of research, organ‐on‐chip technology offers an innovative platform incorporating living human cells in engineered systems simulating key patho‐physiological conditions, such as blood flow and brain mechanical properties.^[^ ^23^ ^]^ These advanced models are particularly valuable for studying the dynamic interactions within the glioma microenvironment, for example, between tumors and immune or endothelial cells, thereby offering detailed insights into cellular behaviors and drug responses under conditions that closely resemble those in humans.^[^ ^24^, ^25^, ^26^, ^27^ ^]^ By integrating these diverse research models, from traditional 2D cultures to advanced organ‐on‐chip technologies, researchers can generate more clinically relevant data. This integrated approach not only enhances our understanding of glioma pathophysiology but also aids in the preclinical assessment of novel therapeutic strategies, accelerating the translation of biomedical discoveries into potential clinical applications. For instance, these models have been instrumental in developing drugs for glioma patients based on the unique characteristics of their tumors.^[^ ^28^ ^]^ In this way, advanced human‐based *in vitro* models are essential for bridging the gap between basic scientific research and practical clinical solutions, driving forward the discovery of new therapeutic targets and the development of effective treatments for glioma patients.

This review article provides a critical analysis of current glioma models and their applications on tumor microenvironment recapitulation and treatment effectiveness with a particular focus on exploring and discussing microfluidic glioma chips including the pathophysiological complexity and associated challenges. Drawing on recent advancements in the field, the review focuses on addressing critical questions posed by clinicians regarding the design of effective glioblastoma models, with a particular emphasis on incorporating BBB and tumor vascularization to enhance their utility in clinical translation.

## 2 Traditional Glioma Research Models

### 2.1 Glioma Cells Used for 2D Models

The 2D cell culture models are the most classic methods for studying drug responses and cellular behavior of glioma, allowing the investigation of cellular processes, the effects of various compounds, and genetic modifications in a simplified and cost‐effective manner. The glioma cells used for 2D culture are mainly immortalized cell lines or primary cancer cells dissociated from tumor tissue. 2D cell models offer several advantages in glioma research, being relatively simple to handle and cost‐effective,^[^ ^29^ ^]^ thus accessible for a wide range of experimental studies. Besides, compared to other models, monolayer cells are cultured in a controlled environment, allowing systematic manipulation of variables. This facilitates the initial screening of drug responses and observation of basic cellular behaviors and mechanisms. The simplicity of the model also enables high‐throughput screening, which is valuable for identifying potential therapeutic agents and understanding fundamental aspects of glioma biology.

The most widely used GBM cells for the 2D cell models are immortalized glioma cell lines such as U87‐MG and U251‐MG. These cell lines are originally derived from glioma tumors but experienced genetic modifications to achieve indefinite proliferation and relatively stable genetic and phenotypic expression after being culture and passage multiple times in the lab conditions. Even though cell lines are more consistent and controllable, they do not resemble the genotype and phenotype of individual patient.^[^ ^30^ ^]^ Due to their genetic stability, they are always used to do high throughput drug screening and development.^[^ ^31^ ^]^

GBM stem cells (GSCs) are stem‐like cells found in hypoxic tumor regions, capable of indefinite proliferation and differentiation into different cell types. The plasticity of GSCs contributes to therapy resistance, making them useful for studying tumor progression and treatment recurrence. Nonstem‐differentiated GBM cells are differentiated from the GSCs and display various phenotypes that contribute to tumor heterogeneity.^[^ ^32^ ^]^ Unlike the GSCs, they have a limited ability to proliferate exhibiting varying degrees of differentiation used to study tumor biology and mechanisms of invasion and metastasis. Although these GBM cells isolated from fresh tumor samples need extensive characterization during the culture process, the advantage is that they represent the features of GBM tumors in patients.^[^ ^33^, ^34^ ^]^

Even though 2D GBM models are easier to handle and widely used for quick drug assessment, they are monolayer cells on a surface without any spatial structure, which are not able to replicate the tissue organization in TME. This model is suitable for studying the direct drug–cell interaction without reflecting the interaction between the surrounding environment and tumor cells. The noncellular component in TME creates a physical barrier in the outside of the tumor tissue and damages the vessel integrity and perfusion around tumor, which increases the difficulty for drug to penetrate the tumor tissues. As a result of that, drug efficacy is always overestimated in the 2D models whereas more advanced 3D models such as GBM spheroids and GoC models frequently reveal limited or absent antitumor activity, better reflecting the challenges of *in vivo* drug delivery and resistance mechanisms. In 2D culture systems, tumor cells are exposed to treatment in the absence of TME, but this overly simplified environment alters gene expression and key signaling pathways, leading to changes in cellular behavior and therapeutic response that often fail to replicate *in vivo* outcomes. For example, glioma cells are associated with numerous genetic mutations *in vivo* but the tumor cell lines have stable genetic and phenotypic expression in culture. Especially for targeted therapy, it often appears highly effective because of the limited genetic heterogeneity and relatively stable mutation profile of GBM cells maintained in 2D culture. However, in the real word, GBM tumors exhibit extensive intertumoral heterogeneity and evolve within a dynamic microenvironment, making it highly unlikely that a single targeted therapy can achieve a durable or complete response. In addition, GBM cells aggressively spread into surrounding healthy brain tissue due to its infiltrative and diffuse morphology, but it is challenging to fully replicate the complex tumor microenvironment through the 2D models. The majority of current 2D culture models only consist of one type of cells lacking the presence of supporting stromal cells, immune cells, and healthy brain cells. This absence fails to recapitulate the complex cellular interactions within the TME, thereby overlooking critical processes such as tumor invasion into surrounding tissue and the development of drug resistance. Consequently, while drug assessment using 2D models can indicate whether a treatment has a direct effect on tumor cells, these models fail to account for the complexity of the TME. As a result, they may not accurately predict clinical drug performance.^[^ ^35^ ^]^ For more comprehensive insights into glioma, these models are often complemented with 3D models, GoC models, or animal models which more accurately mimic the *in vivo* conditions of tumors.

### 2.2 3D Models of Glioma

As mentioned earlier, the 2D GBM models lack spatial structure, cell–matrix interactions, and cellular diversity. In contrast, 3D GBM models closely mimic the *in vivo* TME by preserving tissue‐like structure, enabling physiologically relevant cell–ECM interactions, and incorporating multiple cell types if necessary. While 2D cultures still dominate glioma research because of their reproducibility and scalability, recent studies have highlighted the limitations of traditional 2D cell culture models and more attention has been placed on 3D models due to their high physiological relevance.

The main physiological distinction between 2D and 3D GBM models is the presence of a 3D spatial structure. This tissue‐like structure promotes the natural generation of ECM around the tumor mass and leads to the formation of oxygen/nutrient gradients—features absent in 2D culture. Under the influence of these two parameters, 3D models offer a better representation of how tumors proliferate, divide, and metabolite as a tumor mass. The models are typically divided into three distinct regions: the proliferative zone, the quiescent zone, and the necrotic core. The formation of these regions is primarily driven by the availability of nutrients and oxygen, which diminishes toward the center as the tumor increases in size. Next, the presence of oxygen/nutrient gradients within 3D tumor models contributes significantly to intertumoral heterogeneity by inducing spatial variations in metabolic activity, gene expression, and cell phenotype. Hypoxic regions promote the emergence of therapy‐resistant subpopulations and stem‐like cells, mimicking the complex cellular landscape observed in vivo. Apart from the heterogeneity introduced by the spatial organization of 3D models, a significant source of variability arises from the tumor cells used to establish the models. Tumor cells isolated from patient tissue often carry multiple pre‐existing genetic and epigenetic alterations, contributing to intertumoral heterogeneity. In addition to microenvironmental factors, the cellular source used to generate 3D models plays a crucial role in replicating the TME. Based on the biological sample origin, 3D human GBM models are generated from immortalized GBM cell lines, GSCs, human‐induced pluripotent stem cells (hiPSCs)/human embryonic stem cells (hESCs), and tumor tissues including spheroids, tumorospheres, glioma organoids, tumoroids, organotypic slides, and explants.

#### 2.2.1 Immortalized Glioma Cell Lines Used for 3D Models

Spheroids are the most used 3D models first developed by Carlsson et al. in 1983,^[^ ^36^ ^]^ which are spherical multicellular aggregates generated from established immortalized cell lines with the capability to proliferate indefinitely. Nowadays, spheroids include not only cancer cells but also stromal cells to increase biological complexity. The main advantages of spheroids are homogeneous and easily manipulated, making them suitable for high‐throughput drug screening.^[^ ^37^, ^38^ ^]^ However, the size of spheroids constrains *in vitro* culture duration and, if too large, can adversely affect cell viability and proliferation. Cells on the outer layer of spheroids have better access to nutrients and oxygen,^[^ ^39^, ^40^ ^]^ while cells in the core often experience nutrient and oxygen deprivation, as a result of the formation of a necrotic core. This central necrosis can destabilize the spheroid structure, eventually causing it to collapse from the center after a long period *in vitro* culture. However, the concentration gradations have an influence on the cell activities such as metabolism, proliferation, and drug penetration, creating a more realistic pathological environment.^[^ ^41^ ^]^ For example, Musah‐Eroje et al. demonstrated that glioma was more resistant to the chemotherapies in 3D models compared to 2D models because of the acidity, hypoxic, and abnormal vessels in TME.^[^ ^42^ ^]^ The other advantages of 3D models are the high physiological relevance and better representation of the in vivo drug response; it has been reported that the results of chemotherapies evaluation in 3D glioma conditions align more closely with clinical trial outcomes than those in 2D cells.^[^ ^43^ ^]^

#### 2.2.2 GSCs have the Capability to Self‐Renew and Differentiate in 3D

GSCs play a significant role in the genetic mutations and phenotypic diversity observed in glioma due to their ability to self‐renew and differentiate. This inherent heterogeneity within the tumor promotes drug resistance and facilitates cancer relapse, complicating treatment efforts and hindering long‐term therapeutic success. Singh et al. were the first group to harvest the CD133 positive cells (representing GSC) from tumor biopsies, and these cell subpopulations remained the stem cell features in the *in vitro* culture.^[^ ^44^ ^]^ These GSCs can be grown in 3D tumor‐sphere suspension cultures without any scaffolds, and it has been reported that they maintain the phenotype and genotype of the parental tumor.^[^ ^45^ ^]^ The plasticity of stem cells mainly causes genetic mutations that contribute to glioma relapses, making tumor spheres useful models for studying treatment resistance and cancer progression.^[^ ^46^ ^]^

#### 2.2.3 hiPSCs and hESC Two New Promising Cell Types for 3D Cultures

Glioma does not form a well‐defined tumor mass but rather infiltrates surrounding healthy brain tissue in a diffuse manner in vivo. That is to say, the glioma interaction with healthy brain tissue is an essential component to recreate a more realistic TME. Therefore, hiPSC or hESC differentiation is used to establish neoplastic cerebral organoids (neoCORs) and cerebral organoid‐GBM cocultures (GLICO). In 2018, two groups established the neoCOR through cerebral organoid generation and differentiation via genome editing techniques. Bian et al. used transposon‐ and CRISPR–Cas9‐mediated mutagenesis to edit genes in neural progenitor cells during induction, developing a GBM brain organoid model.^[^ ^47^ ^]^ The other group manipulated oncogenes using CRISPR/Cas9 technology to induce tumorigenesis, and then the tumor cells derived from either brain organoids or patients were cocultured with human cerebral organoids to establish a glioma invasion model.^[^ ^48^ ^]^ To establish the GLICO model, Da Silva et al. coculture the early‐stage cerebral organoids with patient‐derived GBM stem‐like cells to simulate the glioma initiation.^[^ ^49^ ^]^ By starting from hiPSCs and hESC, it is possible to not only represent the interaction between GBM and healthy brain tissue but also mimic the human‐specific genetic and cellular features of glioma, leading to more relevant and accurate insights into the disease development and progress.

#### 2.2.4 Tumor Tissue Explants Maintain the Original Tissue Architecture

Explants, organotypic slices, and tumoroids are all derived from glioma tissue. Patient‐derived explants (PDE) were microdissected into glioma fresh tissue fragments and cultured in Matrigel growing up to 4 mm. It maintains the original tissue architecture and intratumor heterogeneity,^[^ ^50^, ^51^ ^]^ but the successful rate of keeping them in culture *in vitro* is only around 50%.^[^ ^52^ ^]^ Similar to the GLICO model, organotypic slices are composed of glioma cells coculturing with normal brain slices to recapitulate the glioma invasion process.^[^ ^53^ ^]^ It is a powerful model to study tumor migration and invasion because organotypic slices fully reflect the in vivo primary tumor structure.^[^ ^54^ ^]^ Unlike the explants and organotypic slides that maintain the architecture of the tumor tissue, tumoroids are generated from a single cell suspension dissociated from tumor tissue,^[^ ^55^ ^]^ so they tend to exhibit more consistent expression of tumor‐associated antigens (TAA) compared to organoids while reflecting the genetic and molecular profiles of the original tumor.^[^ ^56^ ^]^

Despite the numerous advantages mentioned previously, 3D models also present limitations. While 3D models offer benefits like tumor‐like architecture and accurately predict clinical outcomes compared to 2D culture, they are not as easy to handle as monolayer cell cultures. It takes several hours to days to generate a 3D model because some types of glioma cells lack the intrinsic ability to self‐aggregate or adhere strongly enough to each other, and additional scaffolds are necessary.^[^ ^57^ ^]^ Plus the generation process requests more manual intervention such as matrix preparation, which increases the variability between replication and reduces scalability. Additional manual handling also brings challenges to high‐throughput screening and model standardization. Next, the architecture of 3D models facilitates the formation of nutrient and oxygen gradients within the structure, so it is not easy for the inner layer of cells to get access to nutrients. As a consequence of that, the *in* *vitro* culture time can last up to 21 days due to the formation of a necrotic core in the middle of glioma spheroids.^[^ ^58^ ^]^ Furthermore, imaging 3D tumor models presents challenges, as it is difficult to focus on specific layers or regions within the tumor due to their dense cellular and ECM composition, which limits light penetration and reduces optical clarity, resulting in poor signal detection and image resolution. Unlike 2D cells, which are easily viewed using basic fluorescence microscopy, 3D models require additional clearance step before imaging and advanced techniques like confocal or multiphoton microscopy to visualize internal structures and identify different tumor regions.

In general, 3D tumor models offer a more accurate representation of the structure of glioma in the body and the three regions of the tumor caused by the size provide insights into tumor biology and resistance mechanisms. However, the limited *in vitro* culture time and heterogeneity of 3D models increase the challenges in analyzing and quantifying the models. Moreover, 3D models of glioma only allow to observe the drug–tumor interaction at the organ level. This limitation highlights the need for more advanced platforms such as GoC models or animal models, which aim to provide an in vivo dynamic and systematic environment for the study of systemic processes like disease progression and drug distribution.

### 2.3 Animal Models

Compared to 2D models and 3D models, animal models provide a more systemic physiological environment, enabling a comprehensive understanding of glioma progression and in vivo drug development. Current animal models of glioma include xenograft models, syngeneic models, and genetically engineered models established on mice, zebra fishes, dogs, and fruit flies.^[^ ^19^, ^59^, ^60^, ^61^, ^62^ ^]^ The glioma models on zebra fishes and fruit flies are not commonly used, but zebra fishes can be used to study cancer treatment drugs at large‐scale preclinical trials.^[^ ^61^ ^]^ Fruit flies are useful models to scale up the genetic network screening related to glioma.^[^ ^60^ ^]^

In the patient‐derived xenograft (PDX) model, human tumor specimens or human glioma cell lines such as U87‐MG are implanted into the brain or under the skin of nude mice.^[^ ^63^, ^64^ ^]^ Nude mice lack a fully functional immune system, so it creates a stable environment for human tumor graft to grow and replicate the genomic and phenotypic heterogeneity of primary tumors, making it a valuable platform for screening molecular markers and assessing the personalized treatments.^[^ ^63^ ^]^ However, the limitation is that it is impossible to test the immunotherapies in this model as nude mice have a suppressed immune response due to a lack of T cells.

To include the effects of the immune system, syngeneic models are established by injecting murine glioma cell line GL261 into the intracranial of immunocompetent C57BL/6 mice. With an intact immune system, this model is used to study the tumor‐immune interaction such as the evaluation of checkpoint inhibitors.^[^ ^65^ ^]^ However, the main drawback is that these models rely on the physiological environment of mice which are different species to humans, limiting the applicability of findings.

Genetically engineered and viral vector‐mediated transduction models of glioma are induced by genetic manipulation to introduce mutations in mice such as PTEN and IDH1, which closely represent the key features such as tumor‐related genetic interactions and pathways of glioma in human.^[^ ^66^ ^]^ That makes it possible to monitor the tumor initiation and progress induced by genetic alternations but the tumor development is uncontrollable and does not recapitulate the full genetic and phenotypic diversity after the complex and time‐consuming procedures.^[^ ^59^ ^]^

**Figure 1:**
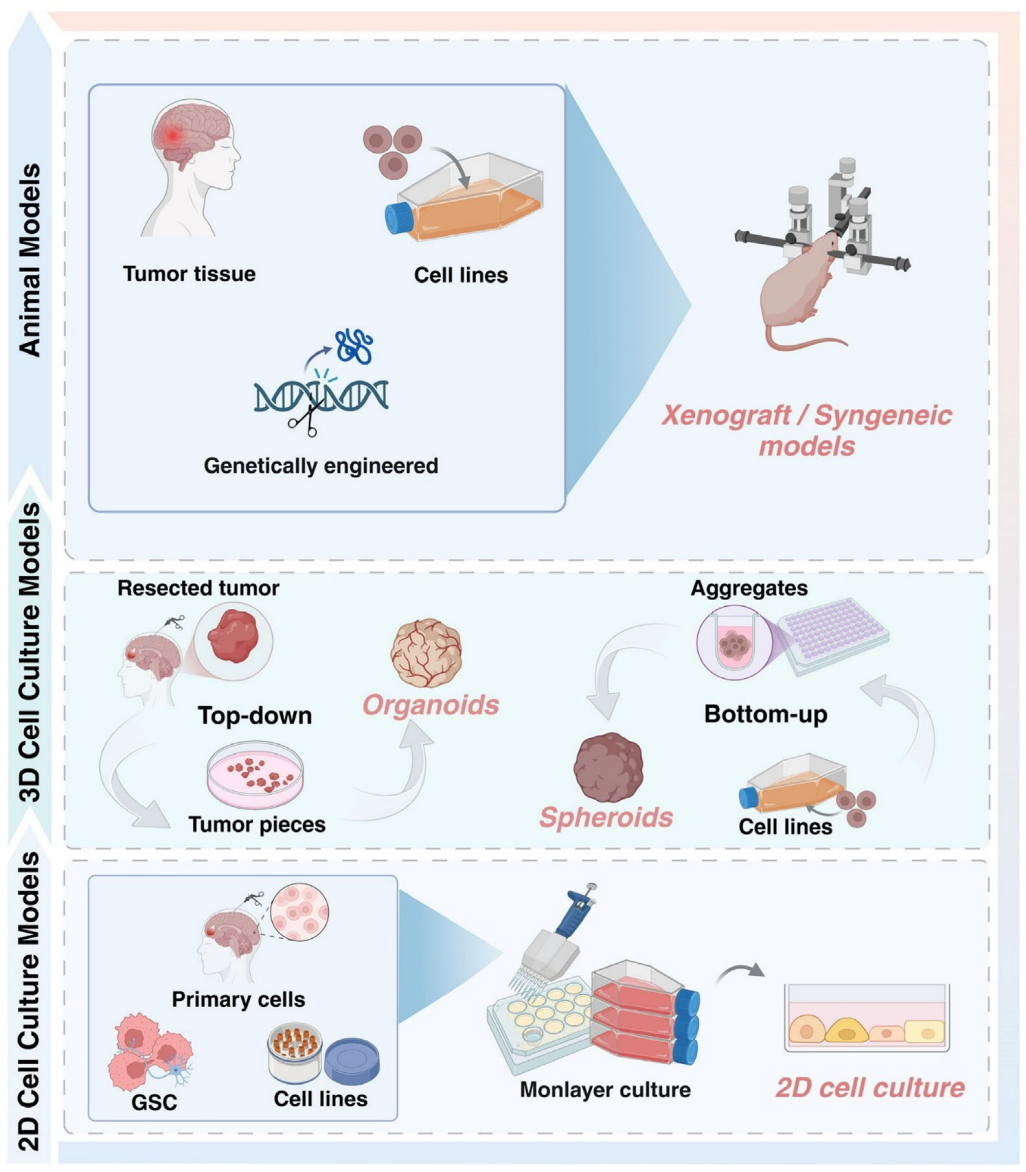
The overview of glioma models. Traditional 2D cell culture models inaccurately predict clinical outcomes, while 3D tumor models like glioblastoma spheroids and organoids better replicate real tumor complexity and heterogeneity, aiding in more effective treatment development. Animal models, though useful, have ethical and biological limitations. However, the gap between 3D and animal models is still quite big since several key factors like perfusion, migration or the dynamics of immune cells, and detachment of potential metastatic cells cannot be properly depicted. Created with BioRender.com.

Even though animal models of glioma allow observation of interaction between tumors and biological systems, systemic pathological environments, and dynamic tumor progress, there are genetic differences between humans and mice so the findings cannot be applied directly to human. Apart from species differences, ethical considerations are another important factor that must be considered. Besides, animal models are more labor intensive and time consuming to develop than traditional *in vitro* models. To solve the issues of species and ethical concern, the GoC model is an alternative method bridging the gap between animal models and 3D models, combining the physiological relevance of *in vivo* systems with the controlled microenvironment of *in vitro* models (**Figure** **1**).

## 3 Microfluidic GoC Models

**Figure 2:**
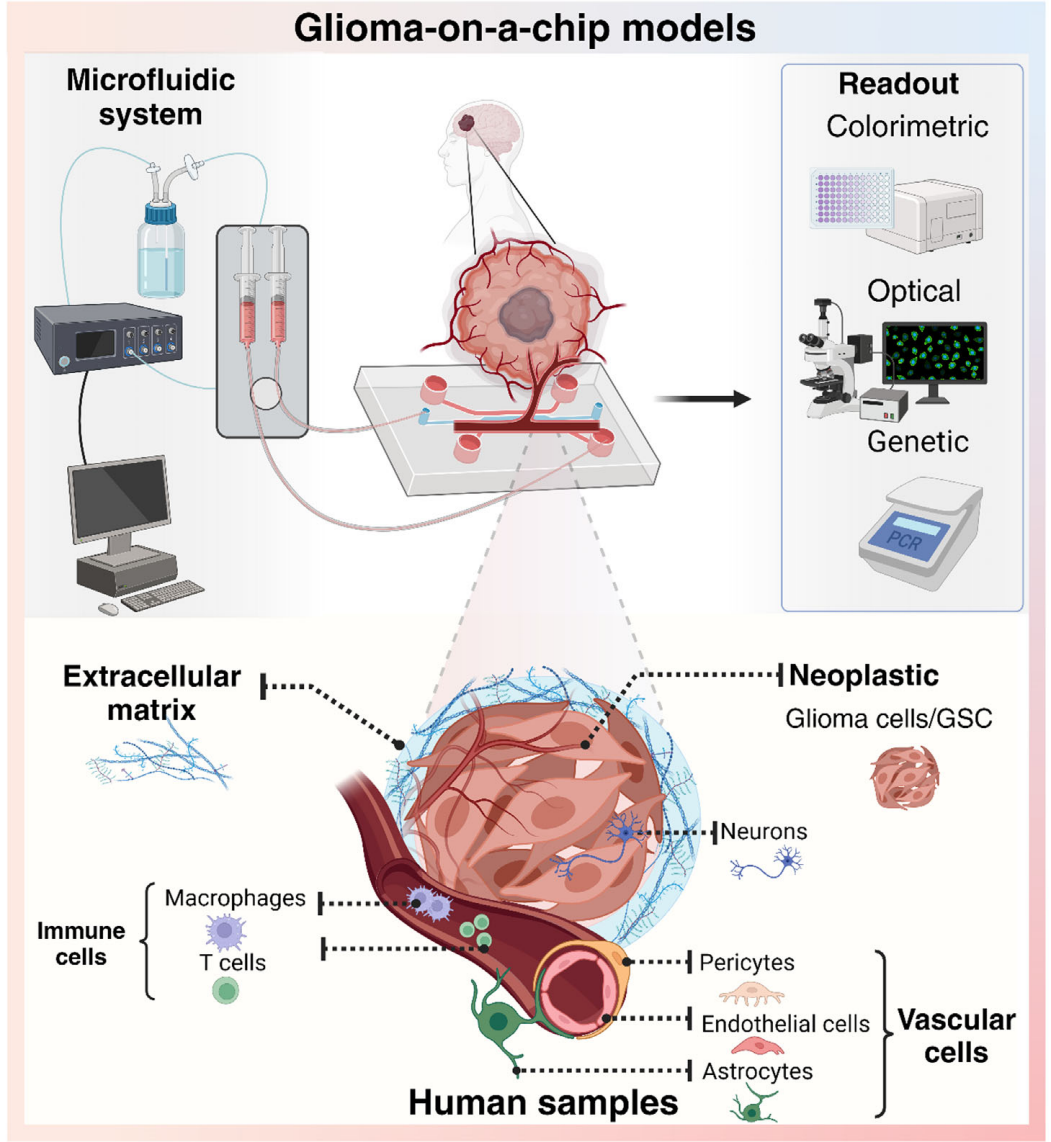
Microfluidic GoC system. Microfluidic system is composed of microfluidic chips, fluidic reservoirs, air pressure pump, drying bottles, and laptop with pump control software. Chip analysis techniques include cell‐based assay, imaging, and external sensor. The main components of glioma model and scaffold cultivating on a chip. Created with BioRender.com.

As a new approach methodology (NAMs), microfluidic GoC models have several advantages over 2D cultures and animal models. This GoC model keeps a balance between improved simulation of human pathophysiology and to certain extent cost‐effectiveness, making them an attractive platform for glioma research. The system typically consists of four main components: the microfluidic system the chip mold fabrication, human biological samples, and ECM^[^ ^67^ ^]^ (**Figure** **2**). Each of these elements plays a critical role in recreating the tumor microenvironment (TME) and facilitating advanced analysis. The GoC allows various analytical techniques to gain deeper insights into tumor dynamics. These methods enable the study of key factors such as tumor progression, invasiveness, and response to treatment, enhancing the application of the GoC model in preclinical and clinical research.

By integrating microfluidic technology, microfluidic GoC models replicate better the dynamic TME at a micrometer scale compared to classic *in vitro* models,^[^ ^25^ ^]^ enabling control over key parameters such as oxygen level and flow speed. The continuous microfluidic flow mimics the pathophysiological blood perfusion, effectively removing waste and delivering oxygen and nutrients to tumor tissue, unlike conventional static cell cultures.^[^ ^68^ ^]^ Next, the shear stress caused by the flow induces specific phenotypes and genotypes of the cells under dynamic culture conditions.^[^ ^69^ ^]^ Based on the direction of the flow in the glioma chip models, the microfluidics is categorized as unidirectional flow and bidirectional flow driven by either gravity or minipump.^[^ ^70^ ^]^ The microfluidic chip was put on a shaker, switching between left and right inclination at a certain angle to enable bidirectional flow,^[^ ^71^, ^72^ ^]^ which is easier to manipulate without connecting the chip to an external pump system. However, in vivo blood flow occurs in a unidirectional manner, whereas the shaker induces bidirectional microflow, which differs from the physiological conditions observed in the body.^[^ ^70^ ^]^ In contrast, the predominant approach to creating microfluidic is to connect to a minipump, which requests complex calculation to ensure the shear stress is the same as that in the vascular system and elaborate handling but the unidirectional flow is more physiologically relevant.^[^ ^70^ ^]^ The addition of the microfluidic system to the glioma models allows a precious controlled setting that better recaptures the in vivo situation of the glioma microenvironment.^[^ ^27^ ^]^ It has been reported that using microfluidic devices to create a dynamic environment can rapidly induce a resistant phenotype of GBM cell line in glioma resistance studies and the transcriptional profile of between in vivo samples and perfusable 3D GBM models has higher similarity in comparison with 2D GBM models.^[^ ^73^, ^74^ ^]^ Establishing a microfluidic environment on the chip significantly affects cell phenotype development, resulting in more physiologically relevant cellular behavior.

Microfluidic chips used in these models are fabricated from various materials, each selected based on bioinertness, fabrication methods, and downstream measurement.^[^ ^23^ ^]^ The widely used materials include thermoplastics like polystyrene, glass, and silicon rubber such as polydimethylsiloxane (PDMS).^[^ ^75^, ^76^ ^]^ Currently, the prevalent material used for the chip molds is PDMS due to the standard microfabrication procedures, ease of operation, and optical transparency,^[^ ^76^, ^77^ ^]^ but the main limitation is the uptake of hydrophobic compounds affecting the evaluation of treatment.^[^ ^78^ ^]^ Alternative materials like thermoplastic polymers and glass are viable,^[^ ^76^ ^]^ but a combination of polystyrene on the top and glass on the bottom may provide an optimal configuration for chip manufacturing, as exemplified by a commercially available MIMETAS chip. The materials used in the chip do not absorb small molecules, and it is easy to obtain high‐resolution images due to the optical transparent glass used for the bottom part.

To comprehensively analyze tumor dynamics on the chip, various methods are employed alongside direct biosensor measurements and optical microscopy observations. Cell supernatant and biological samples can be collected for further analysis at the colorimetric and genetic levels. Biosensors are commonly integrated to monitor key culture parameters, continuously measuring biochemical factors such as pH and oxygen levels, which are critical for maintaining a physiologically relevant TME. Optical techniques, such as fluorescence microscopy, enable real‐time visualization of tumor morphology and invasiveness, providing valuable insights into dynamic cellular behaviors. Meanwhile, colorimetric assays are utilized to assess biochemical markers like cell viability and cytotoxicity, with commonly used methods including MTT or LDH assays. These approaches yield quantitative data on tumor viability and response to treatments under various conditions. Additionally, biological samples, including cells and secreted materials, can be recovered from the chip for downstream analyses. DNA or RNA extraction from these samples allows for genetic investigations, including polymerase chain reaction (PCR), to examine gene expression patterns and mutations. Collectively, these diverse methodologies enhance the ability of GoC models to offer in‐depth insights into tumor progression and therapeutic responses.

The most important component on the chip is the biological sample composed of neoplastic cells and non‐neoplastic cells contributing to the glioma progression.^[^ ^79^ ^]^ Glioma cells and GSC are the critical neoplastic cells that facilitate tumor invasiveness. In principle, 3D or 2D cultures of glioma can be cultivated in a chip‐based advanced *in vitro* system. However, the full potential can be achieved if the most advanced human‐based organoids were integrated into the chip. Other non‐neoplastic cells, including immune cells like macrophages and T cells, stromal cells like fibroblasts and neurons, and vascular cells such as endothelial cells and pericytes, inhibit immune function and promote tumor growth and metastasis.^[^ ^80^, ^81^ ^]^ Plus, the addition of these cells enhances the dynamic interactions between tumor cells and provides a more accurate representation of the characteristics and pathophysiological process of glioma in the brain.^[^ ^82^ ^]^ Moreover, the integration of immune cells into GoC models offers a powerful tool for investigating modern immune‐based therapeutics early in development, such as immune checkpoint inhibitors and adoptive cell therapies. The ability to replicate both the tumor‐invasive behavior and its immune evasion mechanisms within the same platform provides critical insights into tumor progression and therapeutic response, ultimately paving the way for more personalized and targeted treatment strategies for glioma patients.

Last but not least, the ECM acts as a scaffold within the GBM microenvironment,^[^ ^83^ ^]^ providing structural support for cell growth, migration, and attachment. This complex network of proteins and glycosaminoglycans not only facilitates cellular interactions but also plays a significant role in tumor progression. Specifically, the ECM contributes to angiogenesis, the formation of new blood vessels, which is often associated with poor prognosis in glioma patients. Moreover, the ECM creates physical and biochemical barriers that hinder the effective penetration of therapeutic agents into the tumor tissues.^[^ ^56^, ^84^, ^85^ ^]^ Fibronectin and collagen are the two most commonly used proteins to enhance cell adhesion on the glioma chip substrate and emulate the specific features of tumor tissue.^[^ ^86^, ^87^, ^88^ ^]^ Both type I collagen and fibronectin can not only increase the tumorigenic capacity and proliferative properties of glioma cells,^[^ ^89^ ^]^ but also improve the perfused endothelial network formation replicating the BBB in the glioma microenvironment.^[^ ^90^, ^91^, ^92^ ^]^

Considering the fact that the development of a GoC model requires the contribution from multidisciplinary fields like, clinicians, tumor biologist, bioengineers, and microscale fabrication methods.^[^ ^93^ ^]^ The integration of the four perspectives mentioned above is crucial to establish a more pathophysiologically relevant glioma chip and closes the remaining gap between the 3D cultures and animal model nicely. Improving the use of GoC models requires the collaboration between bioengineers and clinicians. Bioengineers provide expertise in optimizing the microfluidic mold, selecting a suitable ECM, and incorporating an advanced microfluidic system, while clinicians can guide the physiological relevance by identifying key tumor features, therapeutic needs, and patient‐specific factors. This multidisciplinary collaboration ensures that the model is both scientifically rigorous and clinically applicable, ultimately leading to the ideal design that better reflects human glioblastoma and improves therapeutic outcomes.

### 3.1 Simplified 3D Glioma Models on a Chip

In recent years, GoC models have emerged as promising tools for studying this aggressive brain cancer and developing personalized treatments.^[^ ^73^ ^]^ To build a more clinically relevant brain cancer model, it is significant to incorporate vascularization, simulate the blood–brain barrier, and include other organs, such as the intestine or liver, in the microfluidic devices.^[^ ^94^ ^]^ Current glioma‐on‐chip models can be categorized based on human pathophysiological relevance, starting from 3D GBM models to 3D multiorgans.

**Figure 3:**
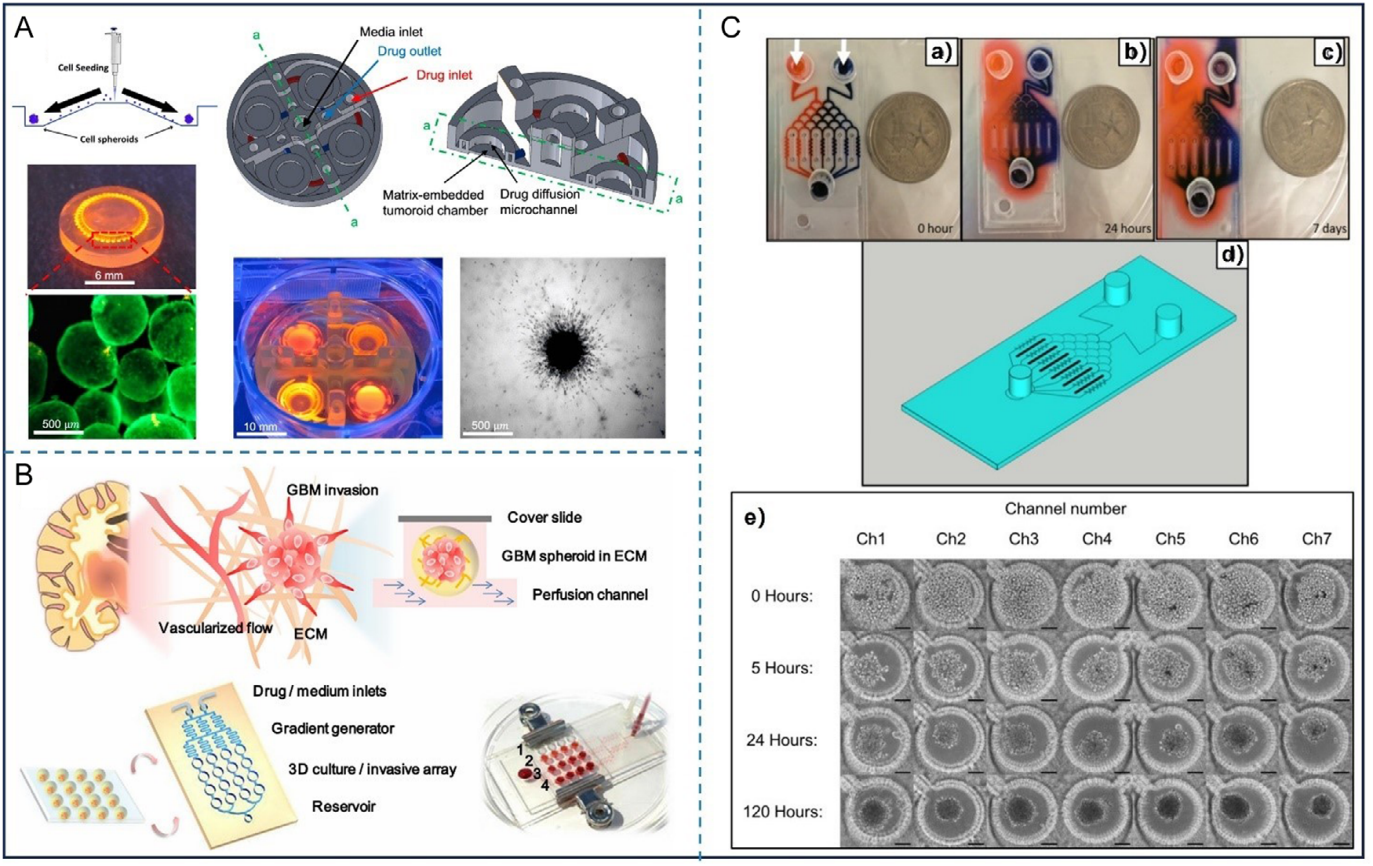
Glioma 3D models on microfluidic chip. A) An *in vitro t*umoroid invasion platform starts with a single cell suspension seeded in a microwell array. Tumoroids are transferred to a tumor‐on‐a‐chip system, featuring four chambers for separate treatments. Then tumoroids in collagen hydrogel are monitored for growth and invasion.^[^ ^101^ ^]^ Reproduced under the terms CC BY 4.0. Copyright 2023, MDPI. B) Using microfluidic chips to simulate the invasive behavior of GBM in a microenvironment *in vitro*.^[^ ^102^ ^]^ Reproduced with permission. Copyright 2018, Springer Nature. C) Two different dyes were added into the inlets to analyze the gradient formed by these two solutions within the microfluidic channels.^[^ ^104^ ^]^ Reproduced under the terms CC BY 4.0. Copyright 2018, Nature Portfolio.

It has been reported that the most commonly used glioma cells to generate 3D glioma models on microfluidic chips are commercial cell lines such as U87‐MG and U251‐MG due to ease of use and the capability to proliferate indefinitely.^[^ ^95^, ^96^, ^97^, ^98^, ^99^, ^100^ ^]^ Meitham et al. developed a GBM tumoroid‐on‐a‐chip model where U251‐MG spheroids in type I collagen to recapture the in vivo ECM surrounding tumor tissue,^[^ ^101^ ^]^ and this model was used to assess the invasive capabilities of GBM spheroids (**Figure** **3**A). In another study, U87‐MG spheroids were embedded in the collagen and cultured under constant low perfusion in a microfluidic device to mimic the dynamic body fluids for drug assessment^[^ ^102^ ^]^ (Figure 3B). Although GBM cell lines, such as those overexpressing biomarkers such as epidermal growth factor receptor (EGFR) and interleukin‐13 receptor alpha 2 (IL13Rα2) are suitable for drug screening,^[^ ^103^ ^]^ they face criticism for not reflecting the specific genotype and phenotype of patients.

Depending on the experiment's purpose, primary glioma cells, tumor tissue, or GSCs have also been used in the glioma chips to enhance patient specificity or tumor heterogeneity.^[^ ^104^, ^105^ ^]^ To select the optimal chemotherapy drug combination for individual patients, GBM spheroids derived from patient tumors were cultured in microfluidic chambers and then different concentrations of chemotherapy drugs were then passed through these chambers to assess drug responses^[^ ^104^ ^]^(Figure 3C). The reason to use patient‐derived GBM cells is that these tumor cells preserve the unique genetic, epigenetic, and phenotypic characteristics of the GBM tumor, including its heterogeneity and intrinsic drug resistance mechanisms. Unlike established cell lines, patient‐derived cells better reflect the actual response of the tumor to specific treatments. This allows for more accurate prediction of therapeutic efficacy, ultimately supporting personalized treatment strategies and improving clinical outcomes. Developing a representative glioma model on a microfluidic chip to investigate the mechanisms or treatments of glioma, it is crucial to select an appropriate source of glioma cells. It means that based on the research purpose, the tumor cell selection should be as complex as needed for the preclinical study, but also as standardized as possible to get a reliable and comparable outcome. Overall, using established cell lines or cells from patient samples is beneficial, as each source provides unique insights into tumor behavior and therapy responses.

### 3.2 Glioma Models Interacted with BBB on a Chip

The BBB is a barrier that keeps the CNS protected by blocking pathogens and toxins from the brain. However, it also restricts drug access to tumors, reducing the effectiveness of antitumor treatments.^[^ ^106^ ^]^ Although drug permeability across the BBB is enhanced in the later stages of cancer due to increased vessel leakage resulting from tumor invasion, it remains crucial to assess the permeability of CNS drugs in preclinical studies to prevent failures during the later stages of drug development.^[^ ^107^ ^]^

**Figure 4:**
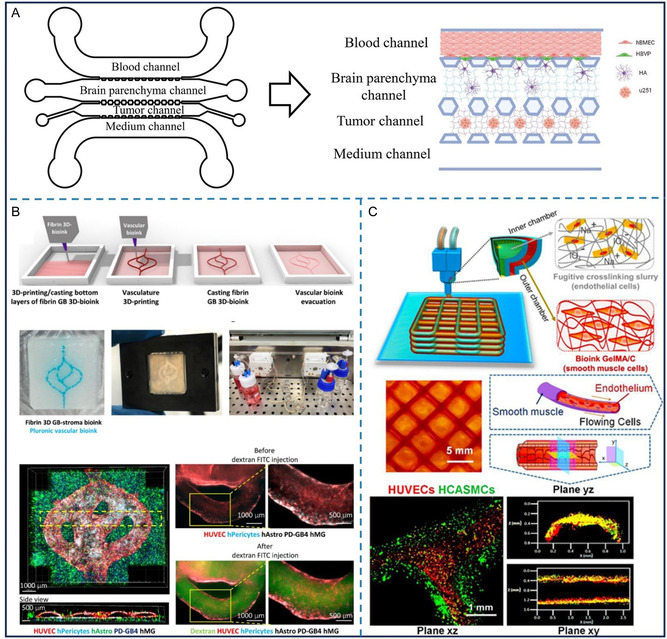
Glioma 3D models with vessel channel on microfluidic chip. A) Shi et al. developed a BBB‐glioma (U251) chip model to assess the permeability and drug efficacy of potential antiglioma components of traditional Chinese medicine. Reproduced with permission.^[^ ^108^ ^]^ Copyright 2022, Elsevier. B) Single brain tumor stem‐like cells mixed with HUVECs and seed in the fibrin gel to establish a GBM perfusable microvasculature‐on‐a‐chip system.Reproduced with permission.^[^ ^115^ ^]^ Copyright 2019, WILEY‐VCH. C) Vasculature structure was printed via 3D bioprinting. Reproduced with permission.^[^ ^111^ ^]^ Copyright 2019, IOP Publishing.

To address this issue, a recent BBB‐glioma microfluidic chip model has been developed, incorporating human brain microvascular endothelial cells, pericytes, astrocytes, and glioma cells. These vascular cells are composed of the *in vitro* BBB with selective permeability used to assess the permeability of the drug candidates before entering the chamber of glioma spheroids. This model demonstrates the *in vivo* CNS drug delivery and evaluates drug efficacy after BBB, highlighting the importance of considering BBB in antiglioma drug development^[^ ^108^ ^]^(**Figure** **4**A). In this study, the BBB function and shear stress in the glioma chip have been characterized in comparison with these parameters *in vivo* to ensure the physiological relevance of the microfluidic glioma chip with BBB. Six Chinese medicine components were assessed on the chip, and it showed that BBB hindered the drug penetration to the tumor site. However, three components still induced tumor cell death on the chip, and the results are in line with the *in vivo* data. The glioma chip with BBB enables the preclinical evaluation of drug candidates based on their BBB permeability and capacity to reach therapeutically effective concentrations at the tumor site, which improves the predictive accuracy of drug screening and helps mitigate clinical failures related to insufficient BBB penetration. However, there are direct contacts between the tumor and the BBB in situ, so the limitation of this model is that the GBM spheroids and the BBB are two separate components of the chip system. Using the 3D bioprinting technique can perfectly solve this problem, and it has been reported that in the printed GBM model, the perfused vascular lumen composed of endothelial cells and pericytes was printed and perfused with the culture medium via the connection to the minipump. The medium passed through the vascular lumen to the patient‐derived GBM spheroids^[^ ^74^ ^]^ (Figure 4B). The transcriptional profile of this GBM 3D model with a perfusable vascular system has a higher similarity to *in vivo* GBM tumors in comparison to GBM 2D models, making it an excellent platform for selecting personalized treatments and identifying new targets. In addition, bioprinting is a powerful technique for fabricating complex vascular structures, showing precise control over geometry and cell location.^[^ ^109^, ^110^ ^]^ Another example is that the development of bioinks promotes rapid crosslinking and cell adhesion, enabling the direct printing of self‐standing vessels with integrated smooth muscle and endothelium. This artificial vessel facilitates perfusion, enabling the flow of fluids, while also mimicking the biochemical properties, such as molecular interactions, and physiological functions, including nutrient transport and waste removal, observed in natural vessels.^[^ ^111^ ^]^(Figure 4C).

**Figure 5:**
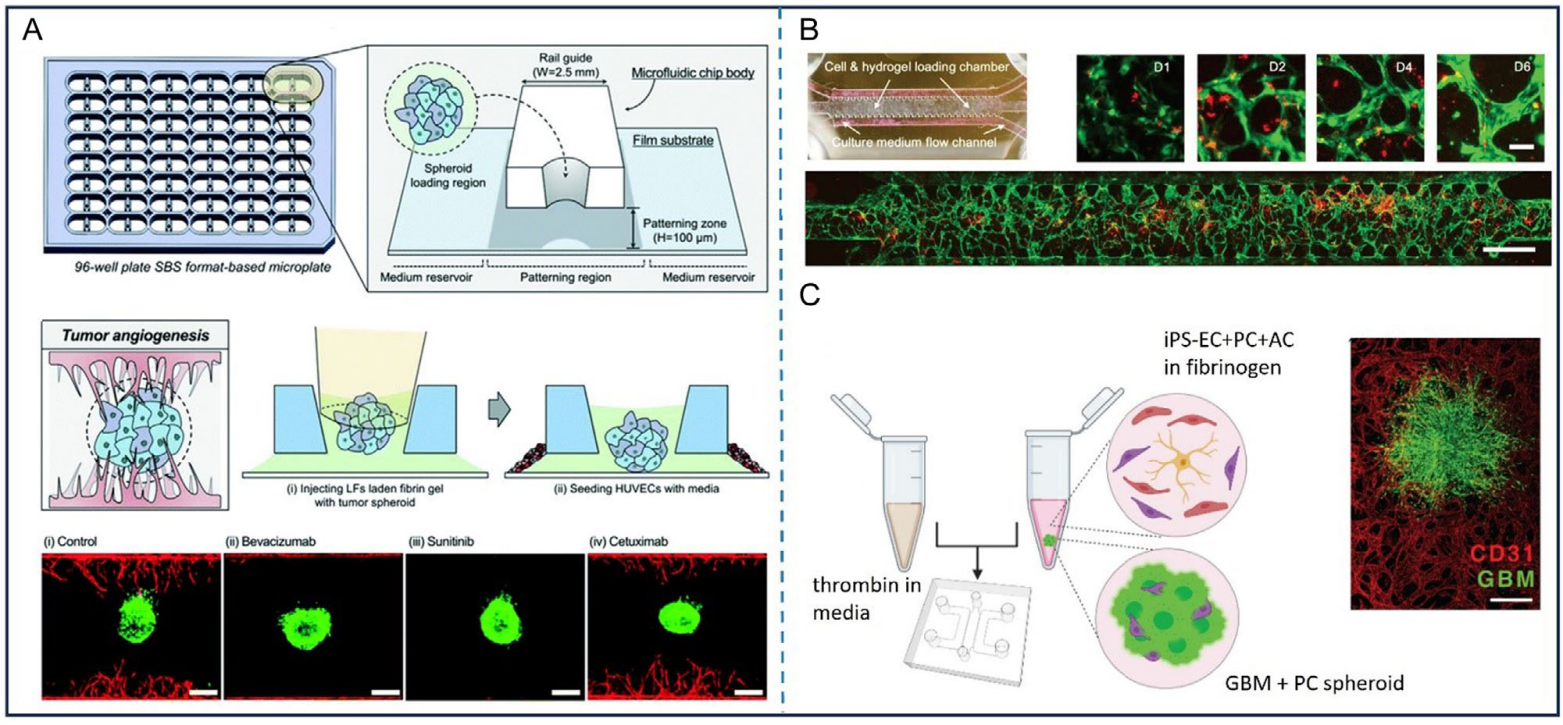
Glioma 3D model with self‐assembly sprouting vessel tube on microfluidic chip. A) U87 spheroids with perfusable blood vessel network on microfluidic chip. Reproduced under the terms CC BY 4.0.^[^ ^111^ ^]^ Copyright 2021, MDPI. B) Single brain tumor stem‐like cells mixed with HUVECs and seed in the fibrin gel to establish a GBM perfusable microvasculature‐on‐a‐chip system.Reproduced with permission.^[^ ^113^ ^]^ Copyright 2019, Advanced Science. C) A vascularized human GBM (GBM22 from PDX) model in a microfluidic device that accurately recapitulates brain tumor vasculature with self‐assembled endothelial cells, astrocytes, and pericytes. Reproduced under the terms CC BY 4.0.^[^ ^114^ ^]^ Copyright 2022, NAS.

Besides glioma models with bioprinted BBBs, recent models feature self‐assembled, perfusable BBBs that more closely replicate *in vivo* condition. The BBB not only incorporates endothelial cells but also pericytes and astrocytes, which spontaneously assemble into the lumen structure of BBB *in vivo* ^[^ ^112^, ^113^, ^114^ ^]^(**Figure** **5**A–C). Xiao et al. demonstrated a microvasculature‐on‐a‐chip model with glioma cells, where endothelial cells were cultured in a fibrin gel formed vascular lumens, spontaneously incorporating patient‐derived stem‐like glioma cells^[^ ^113^ ^]^(Figure 5B). The other example is a GBM spheroid that directly interacts with a spontaneously organized vessel system, including various types of vascular cells on a microfluidic chip^[^ ^114^ ^]^(Figure 5C). In this model, pericytes, astrocytes, and endothelial cells were cultured in the fibrinogen to grow a perfused vascular network, while the glioma cells were mixed with pericytes to pre‐vascularized the tumor model before seeding to the microfluidic chip. It has been developed to incorporate physiological shear stress and allow real‐time visualization. These advanced glioma with BBB models provide valuable platforms for drug screening, exploring GBM mechanisms, and the development of anticancer therapeutics. Although GoC models incorporating BBB represent a significant technological advancement, current research shows that their application remains in preclinical drug screening. These models are often used to identify and exclude drug candidates with low BBB permeability, which may inadvertently contribute to higher failure rates in subsequent clinical trials. As of now, such models have not yet been translated into routine clinical practice.

### 3.3 Glioma Models Integrated on a Multiorgan Chip

**Figure 6:**
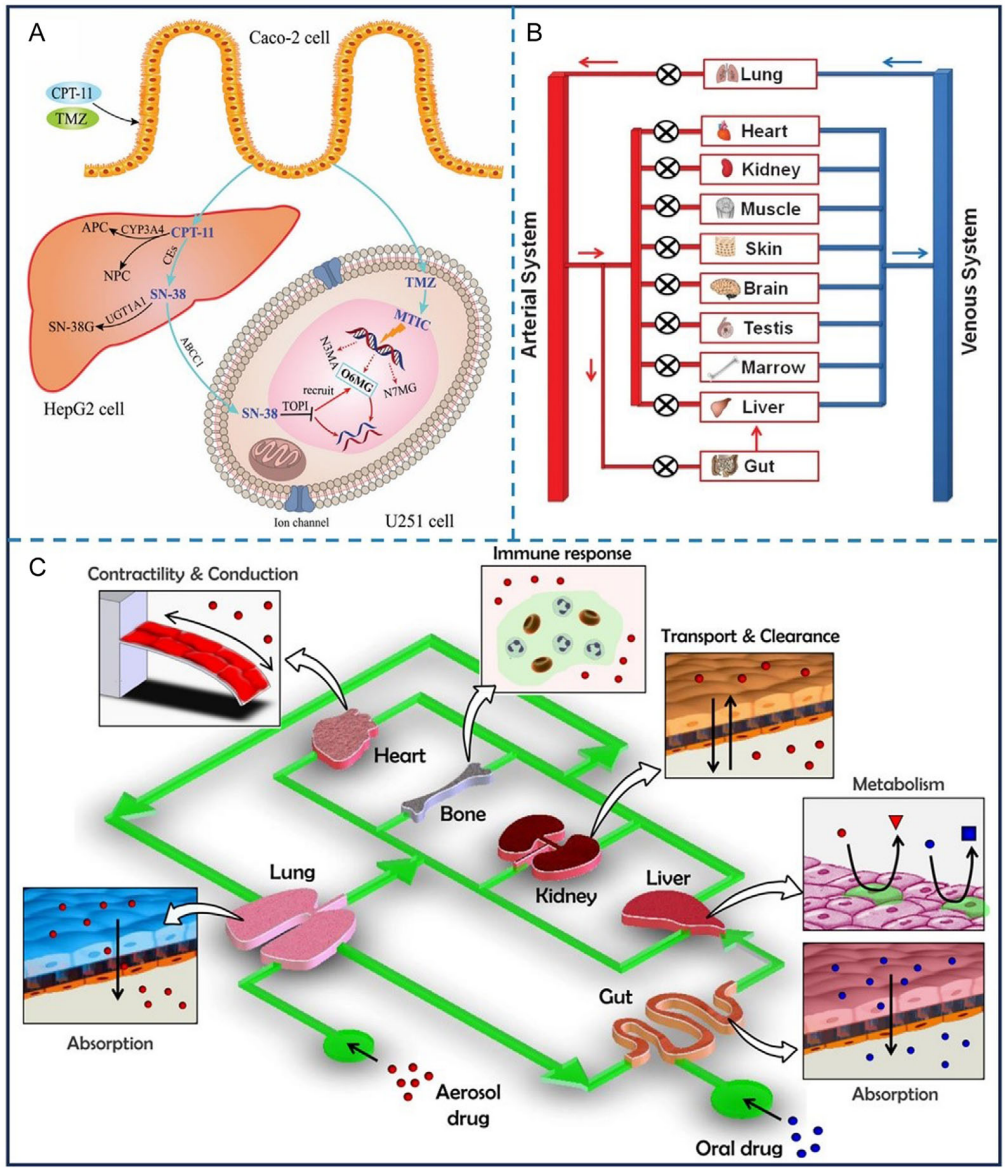
Multiorgans on microfluidic chip. A) This device facilitates the study of CPT‐11 and TMZ metabolism and combinatorial therapy. CPT‐11 is absorbed by Caco‐2 cells and converted to SN‐38 by carboxylesterases and to inactive APC and NPC by CYP3A5.^[^ ^117^ ^]^ SN‐38 is either transferred to U251 cells via ABCC1 or further metabolized to SN‐38G. TMZ is converted to MTIC in HepG2 and U251 cells, where SN‐38 inhibits DNA replication and MTIC induces apoptosis, enhancing CPT‐11's effect.^[^ ^117^ ^]^ Reproduced with permission Copyright 2017, Royal Society of Chemistry. B) Multiorgan chips connected to a microfluidic circulation system.^[^ ^149^ ^]^ Reproduced with permission. Copyright 2012, Royal Society of Chemistry. C) The schematic chart of human‐on‐a‐chip. Reproduced with permission.^[^ ^150^ ^]^ Copyright 2011, Elsevier.

These GoC models mentioned earlier only focus on the cell‐cell interactions within the brain. To investigate drug efficacy and delivery across different tissues,^[^ ^115^ ^]^ multiorgan chips incorporate several cell types and organs, enabling a comprehensive understanding of organ interactions.^[^ ^116^ ^]^ Jie et al. developed an intestine–liver–GBM microfluidic chip that mimics systemic *in vivo* physiological process^[^ ^117^ ^]^(**Figure** **6**A). The intestine simulates drug absorption, while the liver models the crucial process of metabolizing prodrugs into their active forms before reaching the GBM tumor. This model simultaneously monitors drug delivery, metabolism, and therapeutic effects, offering insights not possible with single‐organ models.^[^ ^118^ ^]^ By observing the interaction between the drug, intestine cells, liver cells, and glioblastoma cells, the model offers a more comprehensive view of both therapeutic effects and potential side effects of the drugs. By increasing the physiologically relevance, multiorgan chips have the potential to reduce the reliance on animal testing in terms of drug efficacy and safety assessment^[^ ^119^ ^]^(Figure 6B–C).

In recent years, there has been a significant increase in research and development dedicated to GoC, due to its advantages over conventional monolayer and 3D cultures in recapitulating the *in vivo* pathophysiological environment. The microfluidics platform is capable of anticipating tumor architecture‐related structures, including the BBB and perivascular tumor niches, while simulating physiological shear stress to increase tumor‐environment interactions.^[^ ^94^ ^]^ This application can be employed in both preclinical studies, such as observing cancer cell behaviors, brain drug delivery, and drug screening, and in clinical practices, including personalized treatment selection.

## 4 The Clinical Translation of GoC Models and Their Impact in Clinical Context

**Figure 7:**
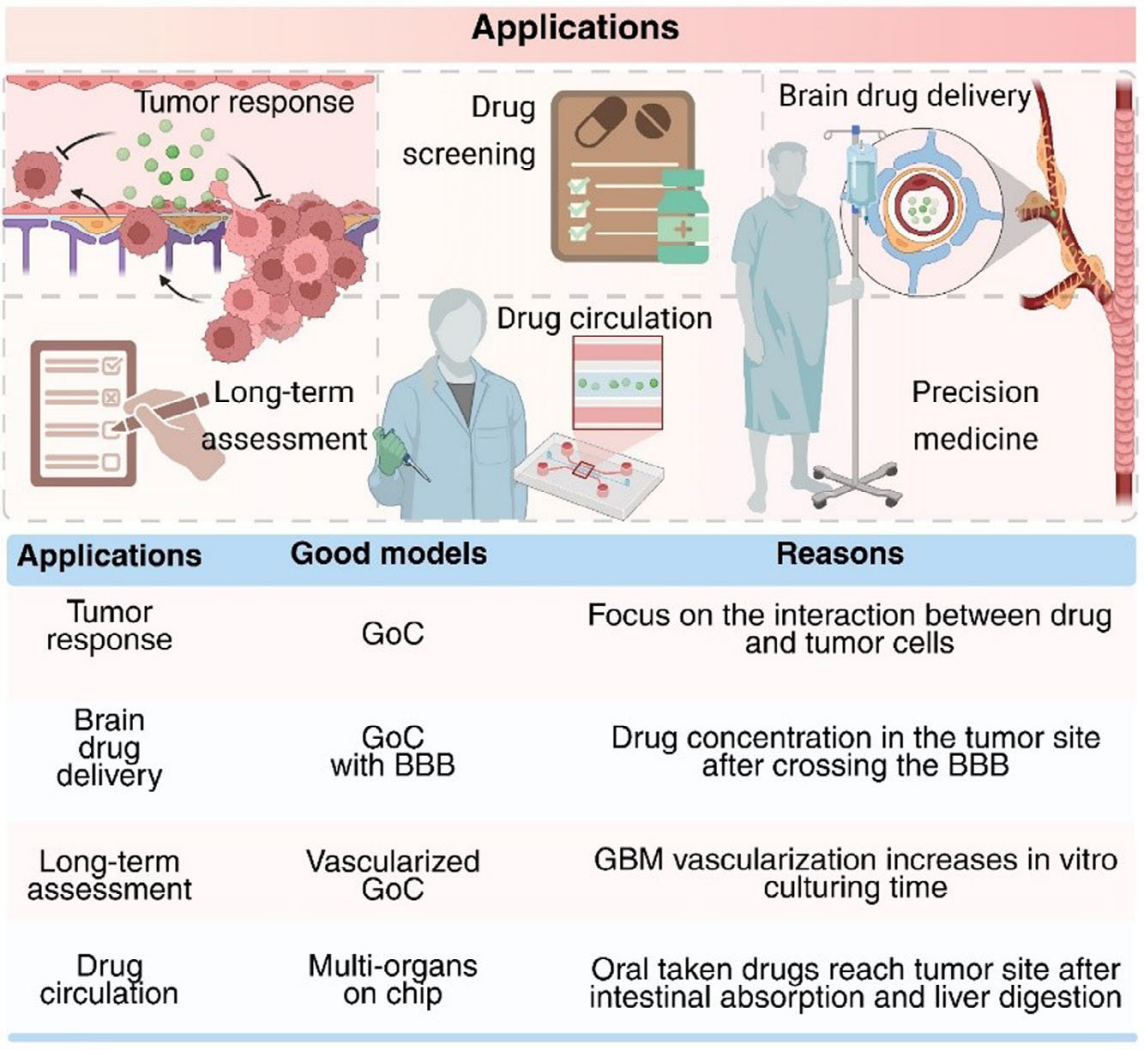
Clinical perspective of GoC usage. (Top) The figure summarizes the main applications of GoC models and (bottom) the most appropriate GoC models based on application. Created with BioRender.com.

The field of organ‐on‐a‐chip technology has made significant progress, driven by the combination of biotechnology and chip engineering. These advances have led to the creation of complex models that closely replicate the biological and physical properties of human tissues. Many of these advancements are frequently highlighted in recent report, reflecting their significant contributions to the advancement of biomedical research (**Figure** **7**). These discoveries drive innovation and impact drug development. However, while these developments highlight the potential of organ‐on‐a‐chip technology, many are mainly driven by curiosity or a focus on engineering innovation. This focus sometimes overlooks key clinical challenges, limiting their practical use in medical applications.

In the subsequent sections, we delve into GoC models that demonstrate the highest potential for addressing the clinical challenges posed by glioblastoma. Our discussion emphasizes the integration of critical elements such as the BBB and tumor vascularization, both of which are vital for accurately simulating the tumor microenvironment and understanding the interaction between drug and TME. Furthermore, we highlight the clinical translational potential of these models, particularly in the realms of personalized medicine and automated high‐content screening assays. By critically evaluating these concepts, we aim to identify and advocate for the GoC designs that most effectively align with clinical requirements, bridging the gap between innovative chip technology and its real‐world applications in glioblastoma treatment and research.

### 4.1 BBB Integration in GoC Models

A major clinical challenge in treating glioma is overcoming the limited permeability of the BBB, which acts as a physical obstacle to delivering drugs to brain tissue.^[^ ^120^ ^]^ Currently, TMZ is the only chemotherapy drug that can effectively cross the BBB and is widely used in glioma treatment. However, its efficacy is limited, and resistance often develops, so it is urgent to find more effective glioma drugs with good permeability. In the preclinical to clinical translation, ineffective drug delivery to tumor sites is a common failure for CNS drugs. Therefore, it is necessary to analyze the BBB permeability for these drug candidates as early as possible in the preclinical phase to avoid the unnecessary waste of expense and resources in clinical trials for drugs with low BBB permeability.

By integrating the complex physiological conditions of the BBB, these glioma‐on‐chip models provide a more advanced physiologically relevant and dynamic environment to assess drug penetration to tumor site and antiglioma activity.^[^ ^100^, ^105^, ^108^, ^121^ ^]^ That is to say, the microfluidic glioma chips accurately replicate the structure and function of the BBB, allowing to study how drugs interact with this barrier in a way that closely mimics *in vivo* conditions. Unlike traditional *in vitro* models, which often fail to account for the selective permeability of BBB, these chips incorporate real‐time monitoring and precise control over environmental factors, offering a more accurate and comprehensive evaluation of a drug efficacy. Yiwen et al. indicated that while the presence of the BBB dramatically reduces therapeutic efficacy of antiglioma drug candidates compared to direct treatment on glioma cells, the resulting data align more closely with *in vivo* finding.^[^ ^108^ ^]^ However, this reduction offers critical insights, as it mirrors the challenges faced in human pathophysiology where the BBB limits drug access to brain tissue. As a result, the data obtained from these models provide valuable information that can better predict clinical outcomes. This predictivity is accelerating the development of new therapies, optimizing drug formulations, and identifying potential treatments that can effectively target glioma within the confines of the BBB.

Next, introducing the BBB in glioma model enhances the accuracy in mimicking the native biological characteristics of the tumor and allows the communication among tumor cells, ECM, vascular cells, stromal cells, and probably immune cells improving the pathophysiological environment of the model. It allows the investigation of the impact of vasculature during tumor progress. Thruong et al. studied the effects of vascularizationon glioma invasion by incorporating a vasculature network that allows cancer cell migration, exploring interactions between the TME and glioma cells and the underlying mechanisms.^[^ ^55^ ^]^ Their work demonstrated that adding a vascular system promotes GSC invasion, maintains its phenotypes, and proved the similarities in tumor invasive behavior between the patient‐derived xenograft model and this microfluidic chip. This article also pointed out that the invasion of GSCs is related to the CXCL12‐CXCR4 signaling. This enables the observation of dynamic tumor responses to therapies, such as changes in tumor proliferation, invasion, metastasis, and molecular signaling pathways, which are crucial for understanding the mechanisms of drug action and resistance.

In a word, including the BBB in glioma models is crucial for enhancing their physiological relevance and providing significant insights into drug permeability and therapeutic efficacy within the brain. While the presence of the BBB reduces the observed therapeutic effects of drugs compared to simplified models, it is closer to *in vivo* data, offering a more accurate reflection of clinical outcomes. Furthermore, integrating the BBB enhances the crosstalk of tumor cells and surrounding cells and the impact of vasculature on tumor progress. Overall, the inclusion of the BBB in glioma models is a useful platform to develop effective treatments for brain tumor.

### 4.2 Tumor Vascularization in GoC Models

Glioma is characterized by high vascularization in clinical, which plays a critical role in tumor progression.^[^ ^122^ ^]^ The vasculature provides a sufficient supply of nutrients and oxygen, supporting rapid tumor cell proliferation and facilitating key processes such as invasion, metastasis, and treatment resistance.^[^ ^123^, ^124^ ^]^ These factors also contribute to the high recurrence rates observed in glioma patients. Incorporating vascularization into *in vitro* glioma models is essential for replicating the tumor microenvironment more accurately, prolonging *in vitro* culture time, tracking brain drug delivery, and studying the impact of tumor vasculature on growth dynamics, therapeutic resistance, and response to treatments.^[^ ^101^, ^125^, ^126^ ^]^ These models provide insight into the role of vascularization in glioma progression and potential therapeutic strategies targeting the tumor‐associated vasculature.

Previous studies reported that necrotic cord started to form in spheroids larger than 500 um, but a solid tumor experiences necrosis once it reaches 4 mm in diameter *in vivo*.^[^ ^127^, ^128^ ^]^ The reason is that the *in vitro* glioma spheroids generally lack the adequate vascularization and the limitation to nutrient and oxygen causes cell death. Implementing tumor vascularization enables the delivery of nutrients and oxygen to the interior of tumor spheroids and extend *in vitro* culture periods. It provides possibility to evaluate the long‐term effects of drug treatments, especially under multiple dosing regimens or treatment combination.^[^ ^101^ ^]^

The inclusion of tumor vascularization in models reflects the infiltration of a vascular network within the tumor mass, closely replicating the environment of drug delivery process observed *in vivo*. This process is composed of the penetration of drug candidates through BBB, their accumulation within the tumor, and subsequent diffusion through the tumor tissue.^[^ ^129^ ^]^ Such a system allows for a more realistic simulation of how drugs reach and distribute within brain tumors. Furthermore, it provides a platform to assess the movement of free drugs and to study tumor cell intravasation and extravasation, key processes involved in tumor progression and metastasis following treatment.

In addition, vascularized tumor in glioma model stimulates the tumor‐associated vasculature under TME, which facilitates a better understanding of tumor‐vasculature interactions. It can be used for the identification of potential tumor‐associated vascular markers and the further development of targeted therapies. One example is Straehla JPP. et al presented a robust *in vitro* GBM model to evaluate the tracking and efficacy of nanoparticles to vascularized GBM.^[^ ^114^ ^]^ The advantage of this model is that GBM spheroids interact directly with perfusable self‐assembled BBB microvessels composed of not only endothelial cells but also pericytes and astrocytes to mimic the human BBB. Through integrating the BBB in the GBM model, it is possible to track the movement of nanoparticles intra/extravasate the BBB toward the tumor site due to the surface functionalization. In addition, involving BBB provides insights into potential tumor‐associated vasculature markers and the tumor‐vasculature interaction.

Incorporating tumor vascularization into glioma models provides several advantages, including a more accurate representation of the hypervascularization typical of glioma *in vivo*. This approach enables the possibility of tumor cells, including those in the core of spheroids, to access sufficient nutrients and oxygen, thus extending the *in vitro* culture period. It also replicates vascular infiltration into tumor tissue, improving the study of drug distribution within the tumor. By promoting crosstalk between tumor cells and surrounding cells, these models offer insights into the role of vasculature in tumor progression. Additionally, they facilitate the identification of tumor‐associated vasculature markers, which can be targeted for the development of more effective therapies.

### 4.3 Clinical Translation of GoC Models

The aim of this chapter is to explore the role of GoC models in clinical translation, focusing on two key aspects: personalized medicine and automated high‐content screening assays. Gliomas, particularly glioblastomas, present significant challenges in terms of treatment and prognosis due to their complex biology and heterogeneity and there is a big variation between each patient. Due to the ability to accurately recapitulate tumor heterogeneity and preserve patient‐specific features, GoC models play an important role in clinical translation. Given the rapid progression of GBM and limited efficacy of the standard treatment, it is urgent to select the most effective drug combination in a short time. The microfluidic environment in the chip not only offers a dynamic physiological relevant environment mimicking the blood flow but also speeds up the drug assessment process. For example, several patient‐derived microfluidic GoC models have been published with the ability to evaluate multiple drug combinations in preclinical studies, which could support clinicians in identifying the most effective treatment regimen for individual patients. In addition, the integration of automated high‐content screening assays into GoC models significantly enhances their utility in drug discovery and personalized medicine. These advanced imaging and analysis systems allow for real‐time, multiparametric evaluation of cellular responses across a wide range of treatment conditions. By combining high‐throughput capabilities, this advanced platform enables rapid assessment of drug efficacy, toxicity, and mechanism of action. This not only accelerates the identification of promising therapeutic candidates but also supports patient‐specific treatment selection in a time‐sensitive clinical context. These two approaches are particularly relevant as they have the potential to bridge the gap between preclinical models and clinical applications, advancing the development of more effective, individualized treatments for glioma patients.

#### 4.3.1 Personalized Medicine

Gliomas are known for their significant genetic heterogeneity, complicating standard treatments which do not work equally well for every patient. To address this challenge, it is essential to develop a platform that represents key tumor characteristics of individual patients and is used to identify the most appropriate personalized treatment option. One approach to achieving this is through the identification of subpopulations within glioma patients, stratified by factors such as genetic mutations, tumor heterogeneity, and treatment response. Stratification strategies can be based on both genotypic profiles such as specific mutations, gene expression patterns, or epigenetic alterations and phenotypic profiles such as tumor morphology and growth patterns.^[^ ^130^ ^]^ These strategies allow for the identification of better responders to therapies within the broader patient population, enhancing the precision of treatment selection. GoC models have emerged as a promising solution in this regard. GoC models have emerged as a promising solution in this regard. By mimicking the unique tumor characteristics of each patient, these chips provide critical insights and recommendations for personalized medical decisions, potentially enhancing treatment efficacy and improving patient outcomes.^[^ ^94^, ^131^ ^]^

Yi et al. created the GBM chip using tumor cells isolated from patients, along with vascular cells and ECM from brain tissues, to replicate the pathological characterization of tumor.^[^ ^132^ ^]^ As expected, the differential drug response observed in the chip is aligned with patient clinical outcomes of standard chemoradiotherapy. This finding highlighted the potential ability of GoC to assist in prioritizing therapeutic options as it effectively captures patient‐specific sensitivities to different drug combinations. To maintain the tumor spatial structure and preserve the patient‐specific features, Horowitz et al. established a microfluidic GBM chip using the organotypic slices from human tumor tissues for multiple drug assessment in preclinical studies.^[^ ^133^ ^]^ This model showed the tumor response to treatment in tumor tissues, but it cannot assess several treatments at the same time. To evaluate the various drug combinations in parallel on models derived from patient tumor tissue, Fan et al. fabricated a chip that evaluates multiple drug treatment conditions simultaneously, featuring two inlets for drug injection at the top, Christmas tree‐shaped channels to generate a concentration gradient of drug combinations, individual chambers for culturing GBM spheroids, and an outlet at the bottom.^[^ ^134^ ^]^ After introducing pitavastatin and irinotecan to the chip, various concentrations of the drug combination were directed to individual culture chambers, and the cancer spheroids were observed at different time points to evaluate the drug response. This study showed a simple and controllable microfluidic chip designed for screening of drug combinations for glioma. This screening process helps in selecting the most appropriate and effective drug combination based on how the tumor responds to different drugs, ultimately aiming to improve the therapeutic outcomes. If patients are resistant to the first‐line glioma treatment, immunotherapy is the other promising treatment that can be used. However, the efficacy of immunotherapy is not predictive due to the immunosuppressive TME and various genetic alterations. To solve this issue, Cui et al. engineered a patient‐derived glioma‐on‐a‐microfluidic‐chip incorporating the cancer cell isolated from patients, microvascular system, and immune cells composed of T cells and tumor‐associated macrophages.^[^ ^135^ ^]^ This personalized glioma chip enables the real‐time observation of the dynamic T cell status and tumor response to the immunotherapy in the heterogeneous and immunosuppressive TME, and it facilitates the patient‐specific biomarker discovery and optimizes the immunotherapeutic treatment to patients.

Even though GoC models are in the beginning stage of fully recapitulating glioma pathophysiology,^[^ ^136^ ^]^ they are emerging as powerful platforms to study aggressive brain cancer, facilitate drug development, and advance personalized oncology. Meanwhile, substantial ongoing research is focused on achieving a more accurate representation of glioma heterogeneity in personalized oncology. In summary, compared to animal models, patient‐derived GoC models offer a distinct advantage by retaining tumor‐specific structural and functional features, as well as clinically relevant genetic mutations. These GoC models improved capture of the complexity of GBM as some features only occur in patients features but are often absent in animal models due to species differences in tumor biology and microenvironment.

Although these findings are currently limited to preclinical studies, they highlight a potential possibility toward clinical application. By enabling drug screening within a tumor microenvironment that mimics key features of glioblastoma including vascularization and tumor heterogeneity, these systems could help guide clinicians in selecting the most effective, personalized treatment regimens. While the routine use of GoC in clinical decision‐making is not yet fully realized, the technology shows clear translational potential. With further validation and integration into clinical workflows, it may serve as a complementary platform to improve treatment precision, particularly for patients with refractory or recurrent GBM. We are convinced that this review helps to raise awareness and stimulate further interest in the development of such models for future clinical use.

#### 4.3.2 Automated High‐Content Screening Assay

Unlike traditional drug screening methods that require extensive manual handling and labor‐intensive processes, microfluidic GoC systems offer a more automated platform for drug evaluation. These systems allow for the simultaneous assessment of multiple drug combinations at varying concentrations within a highly controlled microenvironment. By reducing the need for manual intervention, these platforms not only improve experimental consistency but also significantly enhance throughput, making them highly efficient for high‐content drug screening. Although a fully automated microfluidic chip for drug assessment has yet to be developed, the current advancements suggest that these systems are likely to remain semi‐automated in the near future. By integrating glioma *in vitro* models and automated fluid handling, these chips streamline the testing process, providing a more efficient platform for assessing drug efficacy and optimizing treatment regimens.

Fan et al. fabricated a chip that evaluates multiple drug treatment conditions simultaneously, featuring two inlets for drug injection at the top, Christmas tree‐shaped channels to generate a concentration gradient of drug combinations, individual chambers for culturing GBM spheroids, and an outlet at the bottom.^[^ ^134^ ^]^ After introducing pitavastatin and irinotecan to the chip, various concentrations of the drug combination were directed to individual culture chambers, and the cancer spheroids were observed at different time points to evaluate the drug response. This study showed a simple and controllable microfluidic chip designed for screening of drug combinations for GBM. This example shows that automated GBM microfluidic chip can not only minimize human error and get more reproducible results but also enable high‐throughput screening by processing multiple samples in parallel.

The other significant advantage of microfluidic glioma chips is their ability to enable high‐content screening assays. The integration of biosensors or adaptors into these chips facilitates seamless incorporation into existing workflows, enabling the real‐time collection of data at high speed. This includes monitoring critical parameters such as key metabolites, oxygen levels, and pH during experiments, providing valuable insights into cellular responses.^[^ ^27^, ^137^ ^]^ These real‐time data allow for a detailed assessment of drug dynamics, enabling more accurate evaluation of drug efficacy. Ultimately, this approach supports personalized medicine by efficiently screening and identifying the most effective treatments for individual patient.

In summary, the GBM‐on‐a‐chip systems enable automated high‐content screening assays, significantly enhancing the efficiency and scalability of drug discovery and development. This approach facilitates rapid screening of drug candidates or combinations, allowing for the selection of the most effective treatments. Moreover, the ability to collect real‐time physiological data by installing biosensors or adaptors provides valuable insights into drug efficacy and mechanisms of action, offering a comprehensive platform for advancing personalized medicine and improving therapeutic strategies for glioblastoma.

## 5 Evaluation of the Current Models

**Figure 8:**
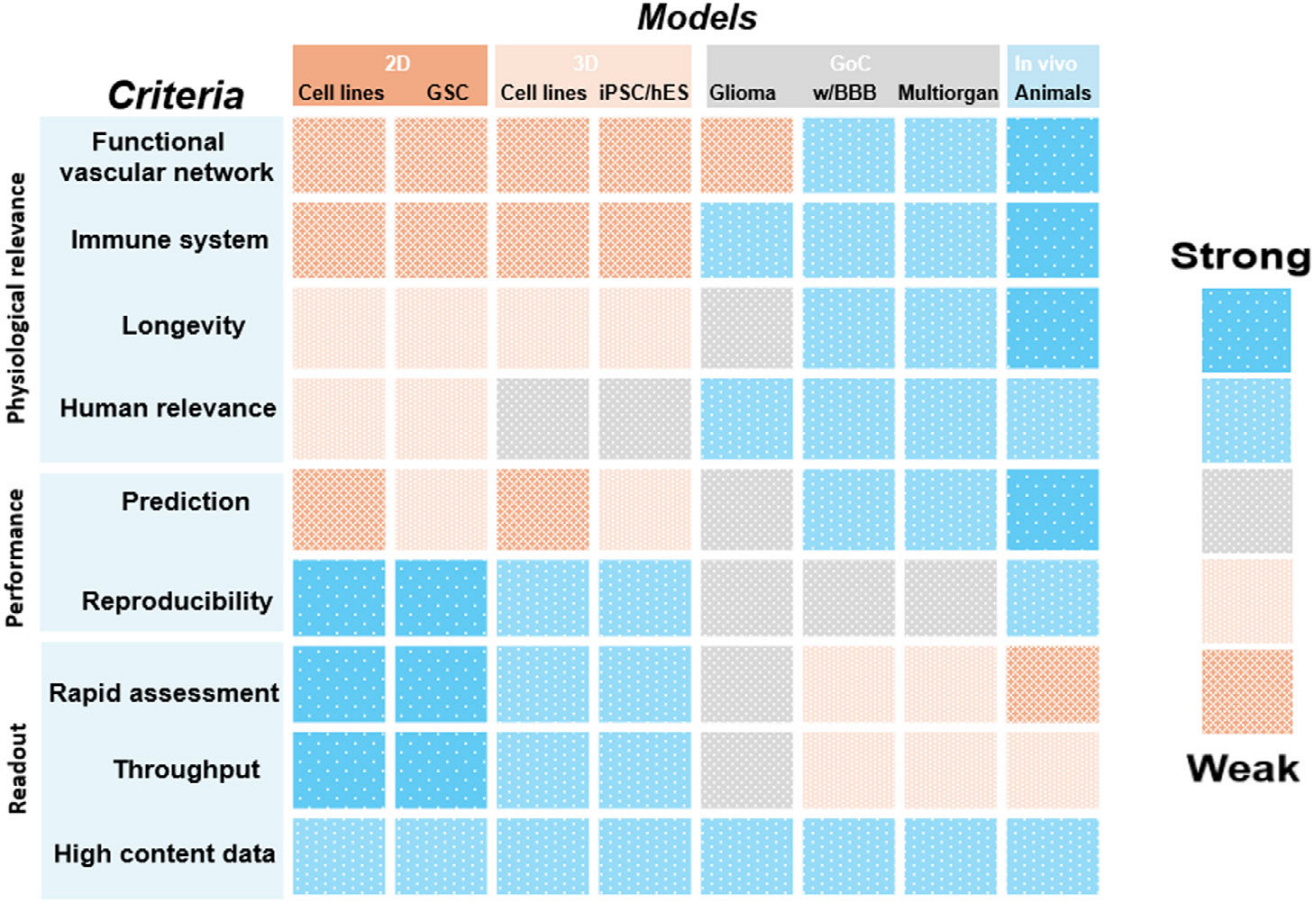
Evaluation matrix of the preclinical GBM models. This heatmap compares glioma models across three criteria: physiological relevance (functional vascular network, immune system, longevity, and human relevance), performance (prediction, reproducibility, and rapid assessment), and readout (throughout and high content data). Darker colors indicate higher relevance or performance, while lighter shades show lower scores. Each cell highlights the strengths and weaknesses of each model, enabling comparative analysis.

The aim of this article is to explore, discuss, and critically evaluate the recent developments of advanced human‐based *in vitro* models, including 2D cell cultures, 3D models, and animal models, with a specific emphasis on the emerging role of microfluidic tumor‐on‐a‐chip models (**Figure** **8**). In general, the main applications of glioma models focus on screening anticancer drug candidates understanding the mechanisms of treatment resistance but also have the potential to preassess and tailor patient‐specific combinatorial treatments. To better reflect clinical outcomes, the current trend in the development of *in vitro* models is to enhance tumor pathological relevance and recreate controlled environments that closely mimic the tumor architecture and the conditions of TME.

Currently, the field of biomedical engineering is advancing rapidly, leading to the development of a wide range of models and hybrid systems, each tailored to address different aspects of glioma research. These diverse models, ranging from microfluidic systems to more complex 3D and animal models, offer researchers the flexibility to select the most appropriate tools for their specific needs, enhancing our ability to answer complex questions and accelerate drug development. While the trend in glioma model development is to closely mimic the TME, this does not mean that more complex models are necessarily better. The guiding principle in model design is that models should be as simple as possible but as complex as necessary to address the specific research goals. Furthermore, it is unrealistic to expect a single *in vitro* model to fully answer all research questions in glioma studies. Depending on the research objectives, even simpler models, such as 2D cultures, can provide valuable insights. In the end, the most effective *in vitro* model is one that balances practicality with the complexity needed, ensuring that the model chosen is best suited to the specific research question.

### 5.1 Model Criteria for Glioma Research

To critically evaluate current glioma models, an evaluation matrix is developed based on three key perspectives: physiological parameters, performance parameters, and readout parameters (Figure 8). The inclusion of these perspectives is essential for assessing how well a model replicates the complexities of TME, evaluating the function as a disease model and analyzing data coming out from the models. This evaluation system represents our interpretation based on the current literature and our own expertise in the field.^[^ ^21^ ^]^ It reflects the perspectives and criteria we consider essential for assessing glioma models.

Physiological parameters focus on how accurately the model mimics the TME, which is crucial for understanding glioma biology. The ability to replicate the TME is assessed through parameters such as the functional vascular network and the immune system—two critical components that significantly impact tumor progression and drug response.^[^ ^138^, ^139^ ^]^ Additionally, tumor longevity and human relevance are evaluated to ensure that the model reflects the long‐term behavior of tumor and their relevance to human physiology. The second perspective focuses on the performance of the glioma models, particularly in their role as disease models for treatment assessment. Key performance parameters include prediction accuracy and reproducibility^[^ ^140^ ^]^ and rapid assessment capabilities. These parameters are selected to ensure that models can reliably predict treatment outcomes, generate consistent results, and enable quick screening of potential therapies. Finally, to accelerate drug development and personalize treatment strategies, the readout parameters focus on data throughput and high‐content data generation.^[^ ^141^ ^]^ The speed at which data is generated, alongside its comprehensiveness, plays a crucial role in informing decision‐making processes.

In the evaluation matrix, a five‐level heatmap is used to assess and compare 2D, 3D, GoC, and animal models across three key perspectives: physiological parameters, performance parameters, and readout parameters. Each level in the heatmap represents a gradation of how well a model meets the evaluation criteria, with the fifth level indicating the strongest match and the first level reflecting the weakest match. For physiological parameters, models that better replicate the complexity of human biology, such as animal models, are rated higher. These systems more accurately mimic in vivo tumor environments, including microvascular and immune interactions, and therefore achieve a fifth‐level rating. Simpler models, like monolayer 2D cultures, receive lower ratings due to their limited ability to reflect the complexity of human gliomas. Regarding performance parameters, the level of match depends on the trade‐off between prediction accuracy and reproducibility. More complex systems, such as animal models, often offer higher predictive accuracy for *in vivo* drug responses and receive a higher rating. However, these systems are also more resource‐intensive, requiring significant time and expertise to establish, which may limit reproducibility. Simpler models, like 2D or 3D cultures, are faster and easier to reproduce but may lack predictive power, resulting in lower ratings. Finally, for readout parameters, simpler models tend to excel due to their faster data generation and straightforward assessments. Monolayer cell cultures, for example, enable quick and high‐throughput analysis, achieving higher ratings in this perspective. In contrast, more complex systems, such as GoC or animal models, may involve time‐consuming setups and analyses, resulting in lower ratings for this parameter. Through this structured evaluation, the heatmap provides a clear and systematic way to assess the strengths and limitations of each model based on their physiological relevance, performance, and readout efficiency. By evaluating glioma models from these three perspectives, researchers can more effectively select the most suitable models to address specific research objectives. Furthermore, the evaluation matrix facilitates a critical analysis of current glioma models, identifying those that not only closely mimic the disease but also provide reliable data to support the advancement of drug development and personalized medicine. The visual presentation of the evaluation matrix relies heavily on the detailed descriptions of glioma models provided in the previous chapters. These earlier sections establish the rationale behind the outcomes by outlining the key features and characteristics of each model. This foundational information ensures that the evaluation criteria, physiological parameters, performance parameters, and readout parameters, are applied consistently and meaningfully across all glioma models.

The evaluation matrix highlights that, overall, animal models emerge as the strongest option across the three criteria of performance, readout, and function. This finding aligns with the current understanding that animal models, with their ability to replicate complex *in vivo* environments, remain a cornerstone in preclinical research. However, it is important to note that GoC models are not intended to completely replace animal models at this stage. Instead, their main role lies in providing a more precise interpretation of *in vivo* data and addressing specific limitations of traditional animal models. While animal models are indispensable for drug assessment prior to clinical trials, they are not universally applicable to all experimental contexts. For instance, T‐cell engagers (TCEs) targeting EGFRvIII on glioma tumors cannot be tested in mouse models due to the absence of EGFRvIII expression in mice. In such cases, 3D BBB model with GBM offers a highly relevant alternative, enabling the evaluation of these targeted therapies in a controlled and human‐relevant microenvironment.^[^ ^142^, ^143^ ^]^ This demonstrates the potential of 3D glioma and GoC models to address plenty of challenges in preclinical research. By bridging the gap between 2D cultures and animal models, they offer an intermediate platform that combines human‐relevant biology with higher throughput and cost‐effectiveness. Consequently, GoC models are poised to play a pivotal role in advancing glioma research, facilitating the transition from *in vitro* studies to animal models, and ultimately to clinical applications.

### 5.2 Remaining Challenges and Limitations

As for all models, the current glioma chip model cannot fully mimic the systemic TME of glioma due to the heterogeneity of tumors and plenty of components in the TME.^[^ ^23^, ^144^ ^]^ Glioma is known to be a heterogeneous tumor, making it challenging to replicate its phenotypes and genotypes *in vivo*. The interactions among the tumor, BBB, cytokines release, and the immune system have an influence on the cancer cell behavior and drug response.^[^ ^145^ ^]^ Even though including the BBB and the microfluidic system can prolong the *in vitro* culture time, the life span of this *in vitro* model is still limited, and it restricts the observation period of the tumor and drug interaction. Plus, the leaking BBB and the ECM are also challenging to reproduce completely due to the patient variability.

Developing the glioma chip system currently requires excessive human intervention, leading to unavoidable variabilities among models and difficulty in maintaining consistency. For example, the generation of glioma spheroids/organoids is mainly carried out by lab professionals especially the organoids derived from stem cells, the ratio of cell population during the differentiation is almost not possible to be the same. Consequently, scaling up model production for high‐throughput drug testing presents challenges in maintaining low variability compared to conventional 2D culture. Plus, the validation process following model establishment to ensure quality is not yet standardized.^[^ ^21^ ^]^

Another significant issue is that establishing a glioma chip system is time consuming. Even though there are some glioma chips available in the market, but these commercial microfluidic chips often do not meet the specific needs of different research groups. As a result, researchers frequently resort to developing in‐house glioma chips, a process that incurs both high costs and considerable time investment. Starting from the chip design, material selection to finally culture 3D tumor model under a precious controlled microfluidic environment requests plenty of optimization and knowledge from different fields, so it requires cooperation with several groups focusing on material sciences, microfluidic engineering, and tumor biology. In addition, the services such as chip molding and materials such as ECM, cytokines, and culture medium for primary cells are all pricey.

In terms of sample analysis, the quantity collected from the microfabricated chip is very small, which may not be well‐suited to current analysis tools. The main used technique to analyze the GoC model is immunofluorescence in many articles because it is not easy to take the glioma spheroids/organoids out of the matrix to proceed with another measurement without damaging it. Even if samples are successfully extracted, their small size makes them prone to loss or excessive dehydration during immunohistochemistry procedures. However, the glioma is quite dense compared to other types of spheroids and organoids, so it creates difficulties to image the tumor embedded in matrix on the chip. In a word, it is necessary to optimize the techniques used to analyze the samples further.

## 6 Conclusion and Future Trends

Compared to conventional *in vitro* model, the GoC offers significant advantages as a powerful platform for rapid assessment of drug candidates or treatment screening in personalized medicine, due to its superior ability to mimic the *in vivo* environment and heterogeneity of the glioma tumor. It has been reported that the accurate rate of using microfluidic chips to predict the survival and recurrence time of glioma patients reaches 86%,^[^ ^146^ ^]^ so the results collected from this model mostly match the patient responses in the clinical practice. Therefore, rather than trying different drug combinations on the patients directly, the treatments can be tested on this model first, and the medical suggestions can be given based on the results in order to speed up the process of finding out the optimal treatments for individual patients.^[^ ^147^ ^]^

The glioma chip model, integrated with the BBB infiltrated into tumor mass, can realistically mimic the environment where GBM tumors are directly connected with a perfusable vascular network.^[^ ^124^ ^]^ It is not achievable with other traditional models. The advantage of integrating BBB is to recapitulate the drug permeability to the tumor site. Plenty of drug candidates are not able to pass through the BBB to reach the tumor site and it is a main failure of CNS drugs in clinical trials. Evaluating the drug candidates on this model before the clinical trials can increase the translation rate from preclinical to clinical by preventing these drug candidates with low permeability from entering clinical trials. In addition, the glioma chip model facilitates the creation of a multiorgan chip system that mimics the dynamic systemic environment and organ interactions *in* *vivo*, providing insight into the long‐term treatment effects and drug‐induced damage to healthy tissues. With the development of this multiorgan system, there is potential to build an *in vitro* human body, which can not only reduce animal testing in the future^[^ ^147^ ^]^ but also significantly accelerate the transition from bench to bedside, enabling the development of more precise, safer, and personalized therapeutic strategies for glioma patients. These models provide a more human‐relevant but ethically favorable platform for research and treatment development, aligning with new ethical standards and facilitating faster scientific advancements. Moreover, enhancing either automated biosensors or advanced imaging technologies in the GoC provides dynamic, noninvasive, and quantitative readouts of real‐time data to have an insight into monitoring cellular interactions, tumor progression, and drug responses at high resolution. When combined with machine learning and multiomics profiling, these approaches could generate high‐content, multidimensional datasets that further our understanding of glioma heterogeneity and resistance mechanisms. Together, these advancements not only speed up the readout but also reduce errors caused by manual intervention.

As GoG models continue to demonstrate their potential in preclinical research, one of the key challenges moving forward is the need for standardization. Currently, the variability in model design, experimental protocols, and assessment criteria limits the ability to compare results across studies and translate findings into clinical practice. To unlock the full potential of GoG models, it is essential to establish standardized guidelines that ensure consistency in key factors such as tumor microenvironment replication, model scalability, and data analysis. Standardization will not only improve the reliability of these models but also facilitate their integration into clinical workflows, enhancing the predictive power of drug testing and personalized treatment strategies for glioma patients.

To enable the use of GoC models in drug approval and medical decision‐making, ensuring reproducibility and standardization in the experimental process is crucial. Developing established protocols will minimize variability between experiments and enhance the reliability of the results. Comprehensive guidelines are needed to set clear requirements and standards for *in vitro* model preparation before entering the market.^[^ ^148^ ^]^ These documents also support regulatory approval processes by providing reproducible and transparent methods for assessing the safety and efficacy of new treatments, ensuring that GoG models can be reliably utilized in clinical settings.

In conclusion, while GoC technology holds significant potential as an *in vitro* model for drug discovery and development, it is essential to acknowledge the challenges that must be addressed during model establishment and data collection. The integration of advanced biomaterials, refined microfluidics, automated workflows, and computational tools presents a promising path to improving the functionality and accuracy of these models. Overcoming these limitations will not only enhance the effectiveness and reliability of GoC platforms but also facilitate their clinical translation, ultimately advancing novel treatment strategies for glioma.

## Conflict of Interest

The authors declare no conflict of interest.

## Author Contributions

**Su Liu**: conceptualization, writing—original draft preparation, graph preparation. **Zhenyu Gong**: conceptualization, writing—original draft preparation, writing—review and editing. **Dairan Zhou**: graph preparation. **Vanesa Ayala‐Nunez**: supervision, project administration. **Tao Xu**: writing—review and editing. **Peter Wick**: writing—review and editing, supervision, project administration, funding acquisition. All authors have read and agreed to the published version of the manuscript.
